# Characteristics of Ag-incorporated bioactive glasses prepared by a modified sol–gel method with a shortened synthesis time and without the use of catalysts

**DOI:** 10.1039/d2ra05671b

**Published:** 2022-10-24

**Authors:** Bui Thi Hoa, Le Hong Phuc, Nguyen Quan Hien, Le Khanh Vinh, Nguyen Anh Tien, Dang Tan Hiep, Vo Thuy Vi, Dang Ngoc Ly, Nguyen Viet Long, Tran Trung Hieu, Le Viet Linh, Nguyen Tuyet Minh, Bui Xuan Vuong

**Affiliations:** Institute of Theoretical and Applied Research, Duy Tan University Hanoi 100000 Vietnam; Faculty of Natural Sciences, Duy Tan University Da Nang 550000 Vietnam; National Institute of Applied Mechanics and Informatics, Vietnam Academy of Science and Technology 291 Dien Bien Phu 700000 Vietnam; Faculty of Chemistry, Ho Chi Minh City University of Education Ho Chi Minh City 700000 Vietnam; Faculty of Chemical Engineering, Ho Chi Minh City University of Food Industry Ho Chi Minh City 700000 Vietnam; Faculty of Food Technology, Ho Chi Minh City University of Food Industry Ho Chi Minh City 700000 Vietnam; Department of Electronics and Telecommunication, Sai Gon University Ho Chi Minh City 700000 Vietnam; Fusi Medical Equipment and Pharmaceutical Joint Stock Company Ngoc Hoa Industrial Park, Chuong My District Ha Noi City 100000 Vietnam; Faculty of Pedagogy in Natural Sciences, Sai Gon University Ho Chi Minh City 700000 Vietnam buixuanvuongsgu@gmail.com

## Abstract

This work presents the preparation of bioactive glasses 70SiO_2_–(26 − *x*)CaO–4P_2_O_5_–*x*Ag_2_O (with *x* = 0, 1, 3, 10 mol%) by a modified sol–gel method with reduced synthesis time based on hydrothermal reaction in a medium without acid or base catalysts. The synthetic materials were characterized by several physical–chemical techniques such as TG-DSC, XRD, SEM, TEM, and N_2_ adsorption/desorption measurement. The analysis data confirmed that the glass sample not containing Ag has a completely amorphous structure, while glass samples containing Ag exhibited a pure phase of metallic nano-silver in the glass amorphous phase. All the synthetic glasses have mesoporous structures with particle sizes of less than 30 nm. The addition of silver to the bioactive glass structure in general did not drastically reduce the specific surface areas and pore volumes of glasses as in previous studies. The bioactivity of the silver-incorporated glasses did not reduce, and even increased in the cases of bioactive glass containing 3, and 10 mol% of Ag_2_O. The biocompatibility of synthetic glasses with fibroblast cells (L-929) was confirmed, even with glass containing high amounts of Ag. Representatively, Ag-incorporated glass samples (sample *x* = 3, and *x* = 10) were selected to check the antibacterial ability using bacterial strain *Pseudomonas aeruginosa* ATCC 27853 (Pa). The obtained results indicated that these glasses exhibited good antibacterial ability to *Pseudomonas aeruginosa*. Thus, the synthetic method in this study proved to be a fast, environmentally friendly technique for synthesizing Ag-incorporated glass systems. The synthesized glasses show good bioactive, biocompatible, and antibacterial properties.

## Abstract 2

This work presents the preparation of bioactive glasses 70SiO_2_–(26 − *x*)CaO–4P_2_O_5_–*x*Ag_2_O (with *x* = 0, 1, 3, 10 mol%) by a modified sol–gel method with reduced synthesis time based on hydrothermal reaction in a medium without acid or base catalysts.

## Graphical abstract

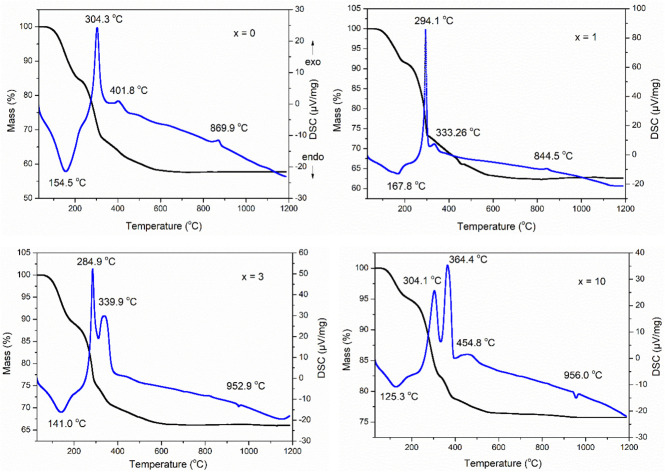

## Introduction

The first bioactive glass was invented by Professor Larry L. Hench in 1969.^1^ The bioactivity of bioactive glass is the ability to form an hydroxyapatite (HA) layer when the material is exposed to biological liquids. This mineral layer has a similar composition to that of human bone, so the damaged bone can be repaired and replaced.^1^ Since the discovery of Larry L. Hench, many scientists have researched and actively developed bioactive glasses by various synthesis methods. Two main synthesis methods widely used for the synthesis of bioactive glasses are the traditional melt-quenching route and the sol–gel route.^2^ The melting method is used to synthesize bioactive glasses by melting a mixture of raw materials at a high temperature (1300–1650 °C) for several hours, and then cooling it in water or in the air.^3^ The melting method is a simple and fast method. However, the high temperature causes the volatilization of the P_2_O_5_ component, resulting in a difference in the synthetic composition. In addition, glasses synthesized by the melting method usually have a dense structure, low porosity, and low bioactivity. On the other hand, the sol–gel method synthesizes the bioactive glasses at lower temperatures. So, it overcomes the limitation of the melting method and has become more popular. Moreover, glasses synthesized by the sol–gel method have higher purity, porosity, specific surface area, and bioactivity than the glasses synthesized by the melting method.^4,5^ In the sol–gel method, some biological beneficial elements such as silver (Ag),^6,7^ copper (Cu),^8,9^ strontium (Sr),^9^ magnesium (Mg),^10^ zinc (Zn),^11,12^ and zirconia (Zr)^13^ were added to synthetic glasses to control the bioactivity and biocompatibility. Moreover, beneficial elements were also incorporated into glass systems to produce new properties; for example, silver is incorporated into glass systems to induce antibacterial properties.^6,7,14–16^ Although the sol–gel method has outstanding advantages over the melting method, it still exits several limitations. Glasses synthesized by the sol–gel method normally take a long time from a few days to several weeks. During the sol–gel process, acids or bases such as HCl, HNO_3_, and NH_3_ solutions are normally used to carry out hydrolysis reactions of precursors, which are harmful to human health and the environment.^2,4^ In this work, for the first time, the modified sol–gel method base on the hydrothermal reaction is used to synthesize Ag-incorporated glass systems. In comparison with the traditional sol–gel method, there are 2 novelty points. The first novelty is that bioactive glasses were synthesized by a green process without any toxic catalysts such as HCl, and HNO_3_. The second novelty is the shortened glass synthesis time.

## Materials and methods

### Synthesis of bioactive glasses

The main chemicals for the synthesis of bioactive glasses are tetraethyl orthosilicate (TEOS, ≥99%, Merck), triethyl phosphate (TEP, ≥99%, Merck), calcium nitrate tetrahydrate (Ca(NO_3_)_2_·4H_2_O, ≥98%, Merck), and silver nitrate (AgNO_3_, ≥99%, Merck). Four glass samples 70SiO_2_–(26 − *x*)CaO–4P_2_O_5_–*x*Ag_2_O (*x* = 0, 1, 3, 10 mol%) were synthesized by the modified sol–gel method based on the hydrothermal reaction. The basic glass system 70SiO_2_–26CaO–4P_2_O_5_ was selected based on previous research.^17^ The composition of the glass systems, as well as the precursors used for the synthesis, are given in Tables 1 and 2. Briefly, the reagents of TEOS, TEP, Ca(NO_3_)_2_·4H_2_O, and AgNO_3_ were added 30 minutes apart, respectively to a mixture solvent of 5 g H_2_O and 40 g C_2_H_5_OH. The reaction system was kept at 70 °C under magnetic stirring at 200 rpm. After the final precursor was added, the reaction system was further stirred for 1 h to form the sol system. The sol system was poured into a hydrothermal reactor, and heated at 180 °C for 24 h. After this time, the wet gel was removed from the reaction vessel and washed thoroughly to neutralize with distilled water, followed by drying the washed gel at 100 °C for 4 h. The dried gel was calcined at about 700 °C (based on the thermal analysis) to make glass systems. It is highlighted that the synthesis method is green without any acid catalyst or base catalyst, and the synthesis time is short compared to the conventional sol–gel method.

**Table 1:** The calculated composition of bioactive glasses 70SiO_2_–(26 − *x*)CaO–4P_2_O_5_–*x*Ag_2_O (*x* = 0, 1, 3, 10 mol%)

Sample	SiO_2_	CaO	P_2_O_5_	Ag_2_O
*x* = 0	70	26	4	0
*x* = 1	70	25	4	1
*x* = 3	70	23	4	3
*x* = 10	70	16	4	10

**Table 2:** Calculated starting chemicals for synthetic glasses 70SiO_2_–(26 − *x*)CaO–4P_2_O_5_–*x*Ag_2_O (*x* = 0, 1, 3, 10 mol%)

Sample	Calculated starting chemicals (g)			
	TEOS	TEP	Ca(NO_3_)_2_·4H_2_O	AgNO_3_
*x* = 0	7.292	0.729	3.070	0
*x* = 1	7.292	0.729	2.952	0.170
*x* = 3	7.292	0.729	2.716	0.510
*x* = 10	7.292	0.729	1.889	1.700

### *In vitro* experiment

The bioactivity was tested by immersing glass powders in simulated body fluid (SBF) according to Kokubo's method.^18^ The SBF solution is synthesized in the laboratory with an ionic composition similar to that of human blood plasma. The ionic composition of the SBF solution is shown in Table 3. The chemicals used for SBF preparation are given in Table 4. Briefly, the SBF solution was prepared in 2 steps. Two separated solutions of Ca–SBF and P–SBF were prepared first, and then these 2 solutions were mixed together to obtain an SBF solution. The advantage of this method is that the SBF solution can be stored for several weeks. In the beginning, 900 mL of distilled water was heated in a thermostatic bath and keep it at 37 °C during the synthetic process. Chemicals in Table 4 were added in turn with an interval time of 30 minutes, and under magnetic stirring. The pH of both Ca–SBF and P–SBF was adjusted to 7.4 (pH of the medium human body) by HCl 6 N solution. The distilled water was added enough to 1000 mL. The solutions of Ca–SBF and P–SSBF were stored in a refrigerator at 5–10 °C for 2–3 weeks. Mixing equal volume of both Ca–SBF and P–SBF solutions to obtain SBF as required. Each glass powder sample with a mass of 250 (g) was immersed in 500 mL of SBF solution (mass/volume ratio of 1/2 mg mL^−1^). The mixture was kept under magnetic stirring at 50 rpm, at 37 °C, for 7 days.

**Table 3:** Ionic composition of SBF solution (10^−3^ mol L^−1^)

Ions	Na^+^	K^+^	Ca^2+^	Mg^2+^	Cl^−^	HCO_3_^−^	HPO_4_^2−^
SBF	142.0	5.0	2.5	1.5	148.8	4.2	1.0
Plasma	142.0	5.0	2.5	1.5	103.0	27.0	1.0

**Table 4:** Chemicals used for synthesize SBF solution

Ca–SBF solution		P–SBF solution	
Reagent	Mass (g)	Reagent	Mass (g)
Tris : C_4_H_11_NO_3_	6.057	Tris : C_4_H_11_NO_3_	6.057
CaCl_2_	0.5549	KH_2_PO_4_·3H_2_O	0.4566
MgCl_2_·6H_2_O	0.6095	NaHCO_3_	0.7056
		KCl	0.4473
		NaCl	16.1061

### Biocompatibility test

The biocompatibility of synthetic glasses was evaluated according to the ISO10993-5 standard as reported in the previous studies.^19–21^ Briefly, the fibroblast cells (L-929) were cultured in 96 well plates (10^3^ cells per well) under standard culturing conditions. Diluted extract solutions (100 μL) of synthetic glasses at various concentrations (0, 12.5, 25, 50, and 100%) were added to cell plates for 24 hours. After that, the media were separated and then 20 μL of MTT solution (5 mg mL^−1^ in PBS) was added. The cell plates were incubated at 37 °C for 24 hours. The media were then discarded and 150 μL of DMSO was added to dissolve the remaining formazan crystals. Finally, the absorbance measurements were carried out at a wavelength of 570 nm using a microplate reader. Four reading times were performed for each sample.

### Antibacterial test

The bacterial strain *Pseudomonas aeruginosa* (Pa) ATCC 27853 obtained from the Department of Microbiology, VNU University of Science, Hanoi – Vietnam, was used to evaluate the antibacterial activity. The antimicrobial testing protocol was developed based on previous research.^22^ The concentration of bacteria was 4.9 × 10^8^ (CFU mL^−1^). The bacterial strain (100 μL) was shaken in 10 mL of medium Lysogeny broth (LB) at 30 °C for 24 h at a shaking speed of 200 rpm. After this time, the medium with high bacterial growth is ready for use. The test glass powders were thoroughly mixed in sterilized distilled water to form a homogeneous suspension following the concentrations of 1; 5; 8; and 10 mg mL^−1^. The bacterial culture medium is spread evenly on the surface of the agar plate, then the agar plate is allowed to dry for 15 minutes. Then, the agar plate was perforated to create well-holes with a diameter of *d* = 4 mm. 50 μL of the glass suspension was added to each well-hole, then the agar plate was incubated in an incubator at 37 °C for 18 h. The result of the antibacterial test was read after 18 h of culture. Testing samples with antibacterial activity exhibit an inhibition zone around well-hole. Water was used for the negative control sample. The antibacterial ability of the test sample was determined based on the ability to inhibit the growth of bacteria, expressed by the diameter of the inhibition zone on the agar plate. Each experiment was repeated twice for repeatability.

### Physical–chemical characterization

The thermal behavior of dried gels 70SiO_2_–(26 − *x*)CaO–4P_2_O_5_−*x*Ag_2_O (*x* = 0, 1, 3, 10 mol%) was analyzed by a Thermogravimetry-Differential Scanning Calorimetry (TG-DSC) on a LABSYS Evo 1600 (SETERAM Instrumentation, France). The measurements were effectuated in dry air at a heating rate of 10 °C min^−1^. The phase composition was verified by X-ray diffraction (XRD, D8 Advance X-ray diffractometer). The samples were measured at a step size of 0.02° in the range of 5°–80° (2*θ*). The elemental composition of glasses was identified by Energy Dispersive X-ray (EDX) method. The textural characteristics of synthetic glasses were investigated by N_2_ adsorption/desorption by using Quantachrome Instruments). The morphologies of glass samples were observed by Scanning Electron Microscopy (SEM, S-4800) and Transmission Electron Microscopy (TEM, JEOL JEM-1400).

## Results and discussion

### Thermal analysis

**Fig. 1:**
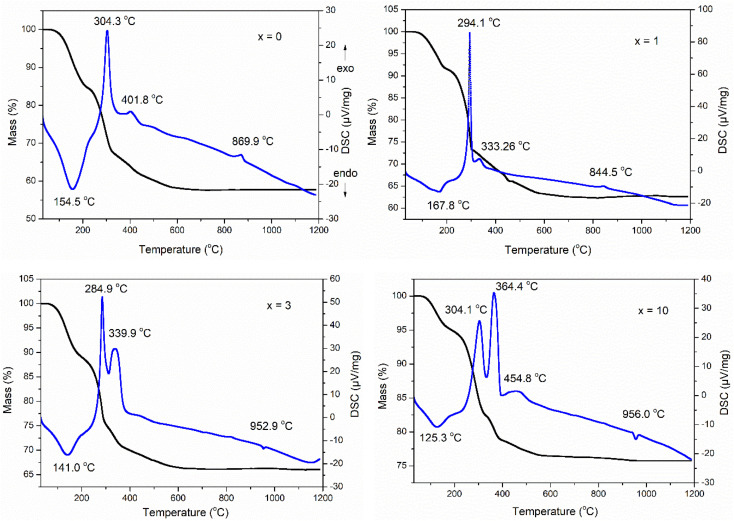
TG-DSC analyses of the 70SiO_2_–(26 − *x*)CaO–4P_2_O_5_–*x*Ag_2_O (*x* = 0, 1, 3, 10 mol%) samples.

The TG-DSC analysis of the as-prepared samples 70SiO_2_–(26 − *x*)CaO–4P_2_O_5_–*x*Ag_2_O (*x* = 0, 1, 3, 10 mol%) is presented in Fig. 1. The first endothermic peak in the temperature range of 120–170 °C associated with the first mass loss in the temperature range of 30–200 °C, was observed in all as-prepared samples. This phenomenon is a result of the thermal removal of free water and alcohol by-products which are generated from the poly-condensation reaction and were not removed during the drying.^23,24^ There are several exothermic peaks in the temperature range of 250–500 °C corresponding to the second mass loss located at around 200–600 °C for all synthesized samples. These exothermic peaks are the results of the combustion of residual organic compounds such as organic precursors.^6^ Interestingly, in all analyzed samples there is no observation of any endothermic peak around 500 °C which is a result of nitrate decomposition and normally appears in the glasses synthesized by the conventional sol–gel method at room temperature.^10,25,26^ This change could be due to the significant differences in synthesis methods. In this study, glass systems were synthesized by the modified sol–gel method based on hydrothermal reaction at high temperature and pressure, while conventional sol–gel glasses were synthesized at room temperature. Thank to strongly reactive conditions in the hydrothermal vessel (high temperature and pressure), the decomposition reactions of nitrate salts can be carried out easily: 2AgNO_3_ → 2Ag + NO_2_↑ + NO↑ + 1.5O_2_↑; Ca(NO_3_)_2_ → CaO + 2NO_2_↑ + O_2_↑. Probably, the decomposition of nitrate salts occurred in the temperature range of 30–200 °C; the mass loss on the TG curve and an endothermic peak on the DSC curve corresponding to the decomposition of nitrates coincide with those corresponding to the removal of free water and alcohol by-products. More, an exothermic peak was observed at 869.9 and 844.5 °C for samples at *x* = 0 and *x* = 1, respectively. This peak is ascribed to the transition phase from the glass phase to the crystalline phase (CaSiO_3_).^10^ It is worth noting that for 2 samples (at *x* = 3 and *x* = 10) this exothermic peak characterizing phase transition completely disappears while an endothermic peak attributing to the melting of the vitreous material system appears at 952.9 and 956.0 °C for the samples *x* = 3 and *x* = 10, respectively.^27^ From the above thermal analysis data, the sintering temperature to obtain the glass systems was selected at 700 °C.

### Phase characterization

**Fig. 2:**
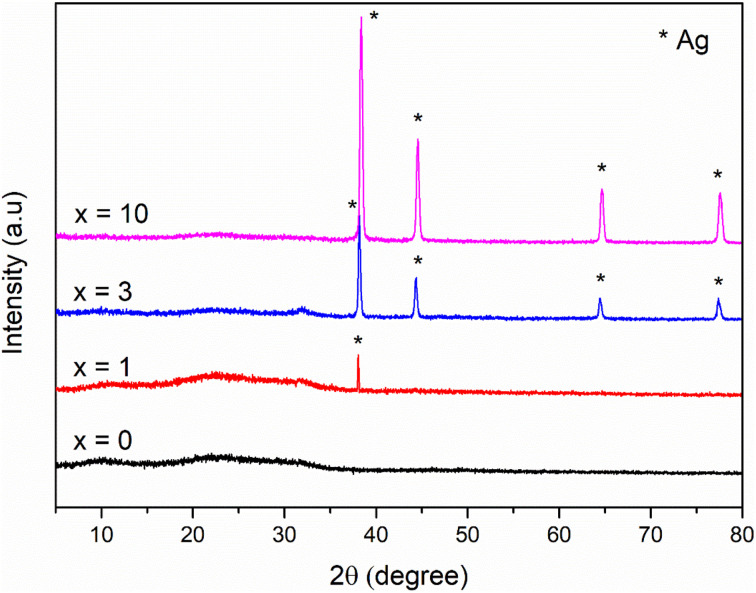
XRD patterns of the 70SiO_2_–(26 − *x*)CaO–4P_2_O_5_–*x*Ag_2_O (*x* = 0, 1, 3, 10 mol%) samples.

Fig. 2 shows the XRD patterns of the synthetic bioactive glasses 70SiO_2_–(26 − *x*)CaO–4P_2_O_5_–*x*Ag_2_O (*x* = 0, 1, 3, 10 mol%). Without containing Ag_2_O (sample *x* = 0), the diffraction patterns showed a wide halo with low intensity, characterizing the amorphous property of synthetic bioactive glass. This result indicated that the non-acid-catalyzed green synthesis method based on the hydrothermal reaction can synthesize an amorphous glass system similar to the sol–gel methods in previous studies.^28,29^ Similarly, the sample *x* = 1 retains the amorphous property of the glass because of the presence of a wide and low-intensity halo. However, a crystalline peak characterizing metallic silver was observed with low intensity. As seen in Fig. 2, by increasing the content of Ag_2_O in the bioactive glass structure, the intensity of the silver peak increased rapidly, which is consistent with the previously reported papers.^7,28^ The existence of metallic silver in glass systems as a single phase in XRD results fits well with TG-DSC analysis where metallic silver is a result of silver nitrates salts decomposition. Additionally, the crystal size of silver was determined based on the Debye–Scherrer equation:^30^
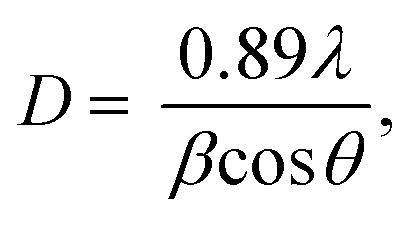
 where *β* is the full-width at half maximum (FWHM), *λ* is the X-ray wavelength; and *θ* is the diffraction angle. The calculation was performed according to four characteristic peaks observed at 38; 44.6; 65; and 77.6 (2*θ*). The mean silver crystal size is about 23.7 nm. Thus, synthetic glass systems contain two phases including the amorphous phase of glass and the crystalline phase of silver metal. These results bring two interesting properties to the synthetic biomaterials, the glass phase will show its bioactivity, besides, the existence of nano-Ag elements will exhibit antibacterial properties.

### Elemental analysis

**Fig. 3:**
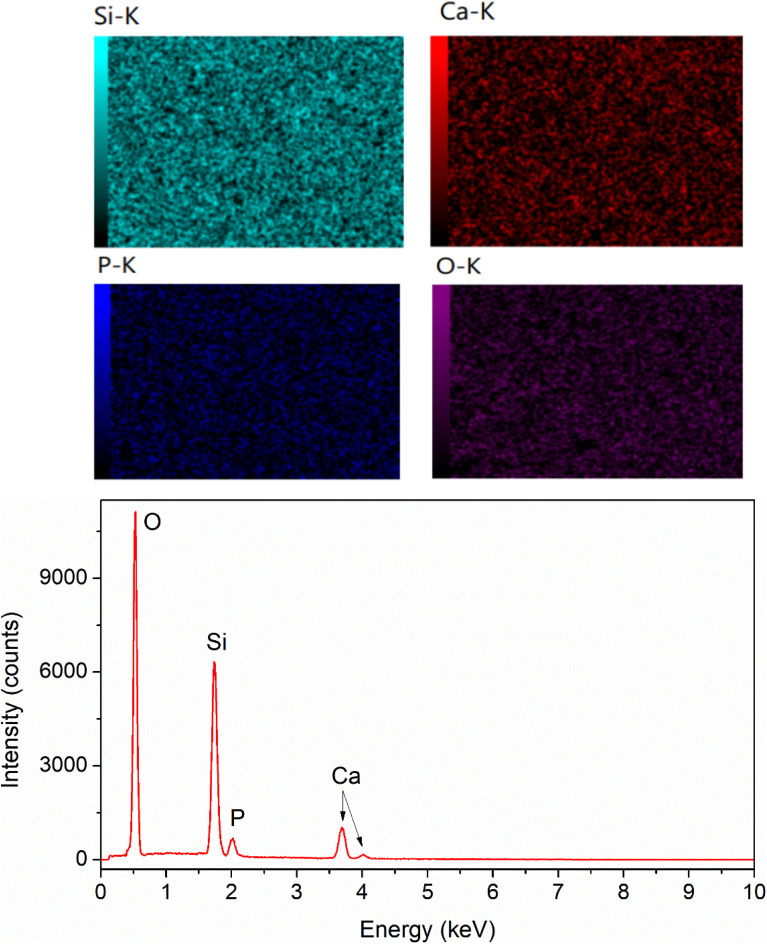
EDX mapping of glass sample *x* = 0.

**Fig. 4:**
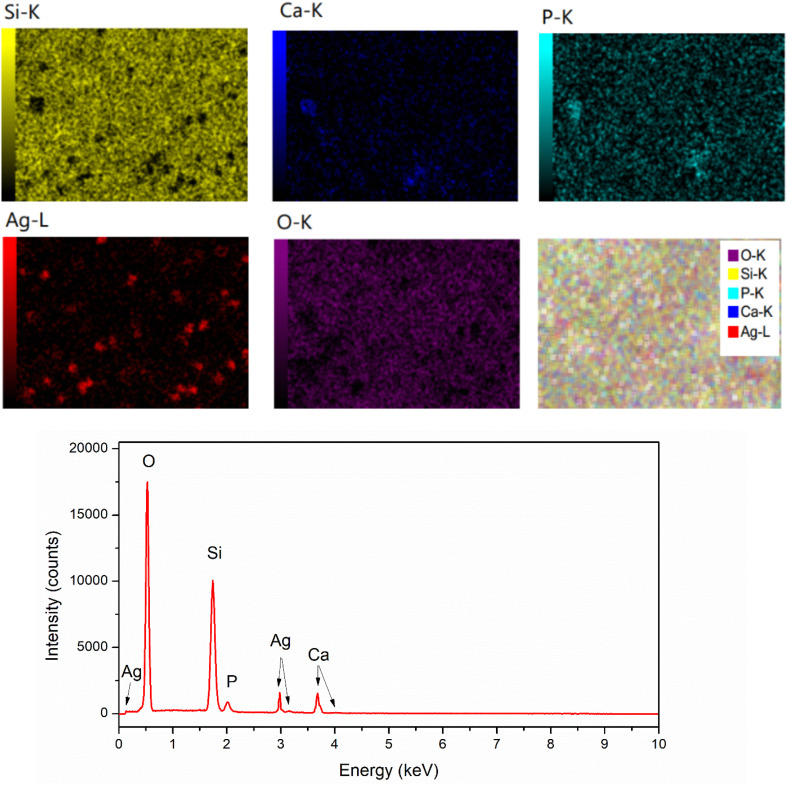
EDX mapping of glass sample *x* = 10.

To confirm the existence of Ag in the synthetic glasses, EDX analysis was performed for two representative samples (sample *x* = 0, and *x* = 10). The obtained mapping images and spectra are presented in Fig. 3 and 4. For sample *x* = 0, the obtained data represents the appearance of four elements Si, Ca, P, and O (Fig. 3). However, sample *x* = 10 shows the presence of Ag in its composition (Fig. 4). This result confirms the success of Ag-addition in the glass network. The elemental composition of synthetic samples is presented in Table 5. The obtained data show the agreement of the elemental composition analyzed by the EDX method compared with the calculated theoretical values.

**Table 5:** Elemental composition of synthetic glasses

Element (%)	Sample *x* = 0		Sample *x* = 10	
	Calculated theoretical value	EDX analysis	Calculated theoretical value	EDX analysis
Si	24.14	23.56	23.33	20.23
Ca	8.96	9.25	5.33	6.91
P	2.76	2.58	2.67	2.60
O	64.14	64.61	62	63.81
Ag	0	0	6.67	6.45

### Textural investigation

**Fig. 5:**
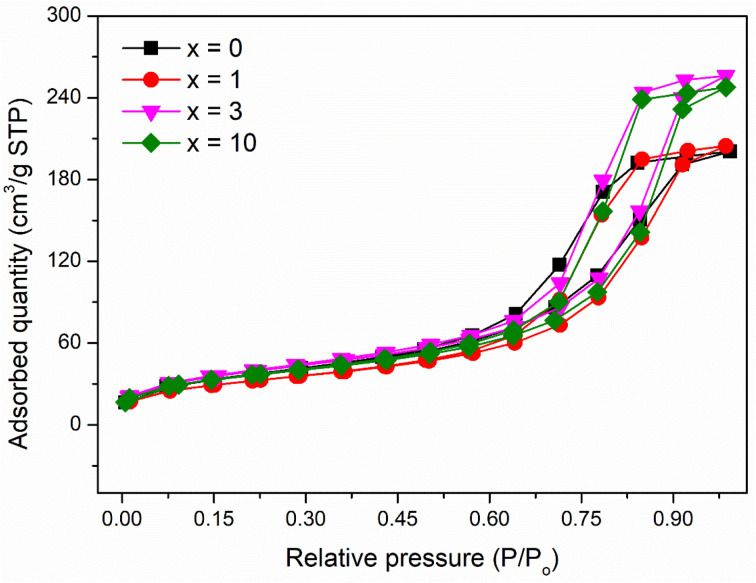
Nitrogen adsorption/desorption isotherms of the 70SiO_2_–(26 − *x*)CaO–4P_2_O_5_–*x*Ag_2_O (*x* = 0, 1, 3, 10 mol%) samples.

**Fig. 6:**
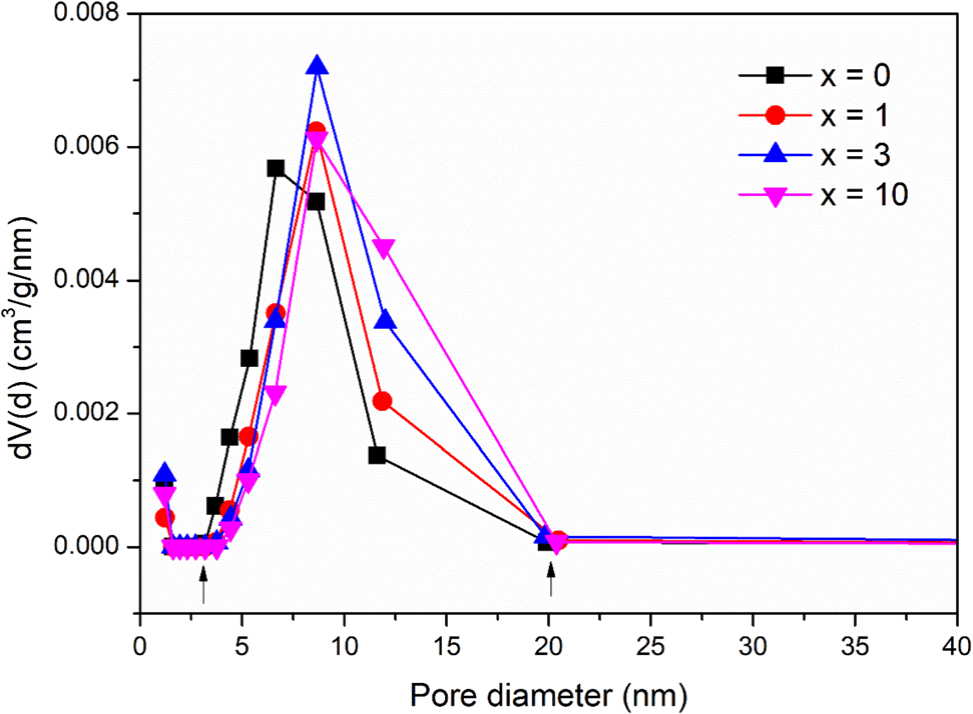
Pore size distribution of the 70SiO_2_–(26 − *x*)CaO–4P_2_O_5_–*x*Ag_2_O (*x* = 0, 1, 3, 10 mol%) samples.

The pore structure of synthetic glasses has been studied using a Quantum Instrument. Glass samples were degassed to remove moisture from the pores, and then analyzed by N_2_ adsorption. The adsorption cross-section of the adsorbed N_2_ molecule was taken as 0.162 nm^2^ to calculate the specific surface area according to ISO 9277 standard. The pore volume is obtained from the amount of N_2_ absorbed by the testing sample in the range of relative pressure (0.9947 < *P*/*P*_0_ < 0.9956). The mean pore diameter was determined by the formula: *d* = *V*_p_/*S*_BET_. Fig. 5 presents the results of textural analysis of synthetic samples 70SiO_2_–(26 − *x*)CaO–4P_2_O_5_–*x*Ag_2_O (*x* = 0, 1, 3, 10 mol%). According to IUPAC classification, the isotherms obtained from the samples are type IV, implying that each of these samples contains a mesoporous structure with pore diameters between 2 and 50 nm;^31^ and the isotherm curves exhibit the type H1 hysteresis loop, corresponding to the ordered structure with the large pores of silica mesoporous materials. The pore size distributions of synthetic samples are shown in Fig. 6. The pore sizes vary between 3 and 20 nm for all synthesized samples and the median pore diameters are mainly in the range of 6.7 to 8.7 nm. The textural data for pore volume, median pore diameter, and BET surface area are reported in Table 6. The interesting aspect of this table is that the specific surface area and pore volume of the silver-containing samples varied slightly, and even increased compared with the silver-free sample. In particular, Ag-containing glasses have much higher values of pore volume and specific surface area than previous Ag-containing conventional sol–gel glasses.^8,29^ In high contrast, the previous studies on the synthesis of Ag-incorporated glass systems by the conventional sol–gel method reported that the specific surface area and pore volume of the synthetic glasses decreased drastically when the silver concentration increased gradually and this could be the result of the potential presence of Ag nanoparticles that were deposited on the pore walls and blocked the pores.^8,29^ Synthesis of bioactive glass systems by the conventional sol–gel method normally takes a long time-consuming, especially from sol state to gel state. Therefore, the poly-condensation process can lead to the diffusion of silver ions getting deep inside and tightly binding the pores and gaps of the gel system. The heating stage of dried gel will release metallic silver and fill up the pores of the material system, resulting in a drastic decrease in pore volume and specific surface area. In this work, the bioactive glasses systems were synthesized by the modified sol–gel method based on hydrothermal reactions in a short time and without catalysts. The hydrolysis of precursors was carried out in a hot solvent system without using catalysts, while the gelation was performed under vigorous reaction conditions (high temperature and high self-pressure) in the hydrothermal system.

**Table 6:** Textural data of the 70SiO_2_–(26 − *x*)CaO–4P_2_O_5_–*x*Ag_2_O (*x* = 0, 1, 3, 10 mol%) samples

Samples	Pore volume (cm^3^ g^−1^)	Median pore diameter (nm)	BET specific surface area (m^2^ g^−1^)
*x* = 0	0.33	6.7	127.6
*x* = 1	0.34	8.6	113.0
*x* = 3	0.42	8.7	137.4
*x* = 10	0.40	8.7	126.1

Moreover, TG-DSC results (Fig. 2) consolidated the interesting results in the textural analysis (Table 6). The above TG-DSC results indicated that the formation of metallic silver differs completely from the formation of metallic silver in the calcination period as shown in the conventional sol–gel synthesis. The synthesis process in this study is different in terms of sol, gel, and nano-silver formations compared with the conventional sol–gel method. The synthesis process was carried out first by stirring the precursors in the solvent at 70 °C. The high temperature accelerated the hydrolysis reaction without the use of any catalysts. Additionally, vigorous reaction conditions in the hydrothermal reactor in the next step (high temperature and high self-pressure) accelerated the sol polymerization, and the sol–gel transition was performed in a shorten time. Therefore, based on the textural analysis, the silver incorporated bioactive glasses synthesized by the improved sol–gel method in this work, probably preserve their pore volume and specific surface area, which are important factors affecting the bioactivity of glass materials.^32^

### Morphology observation

**Fig. 7:**
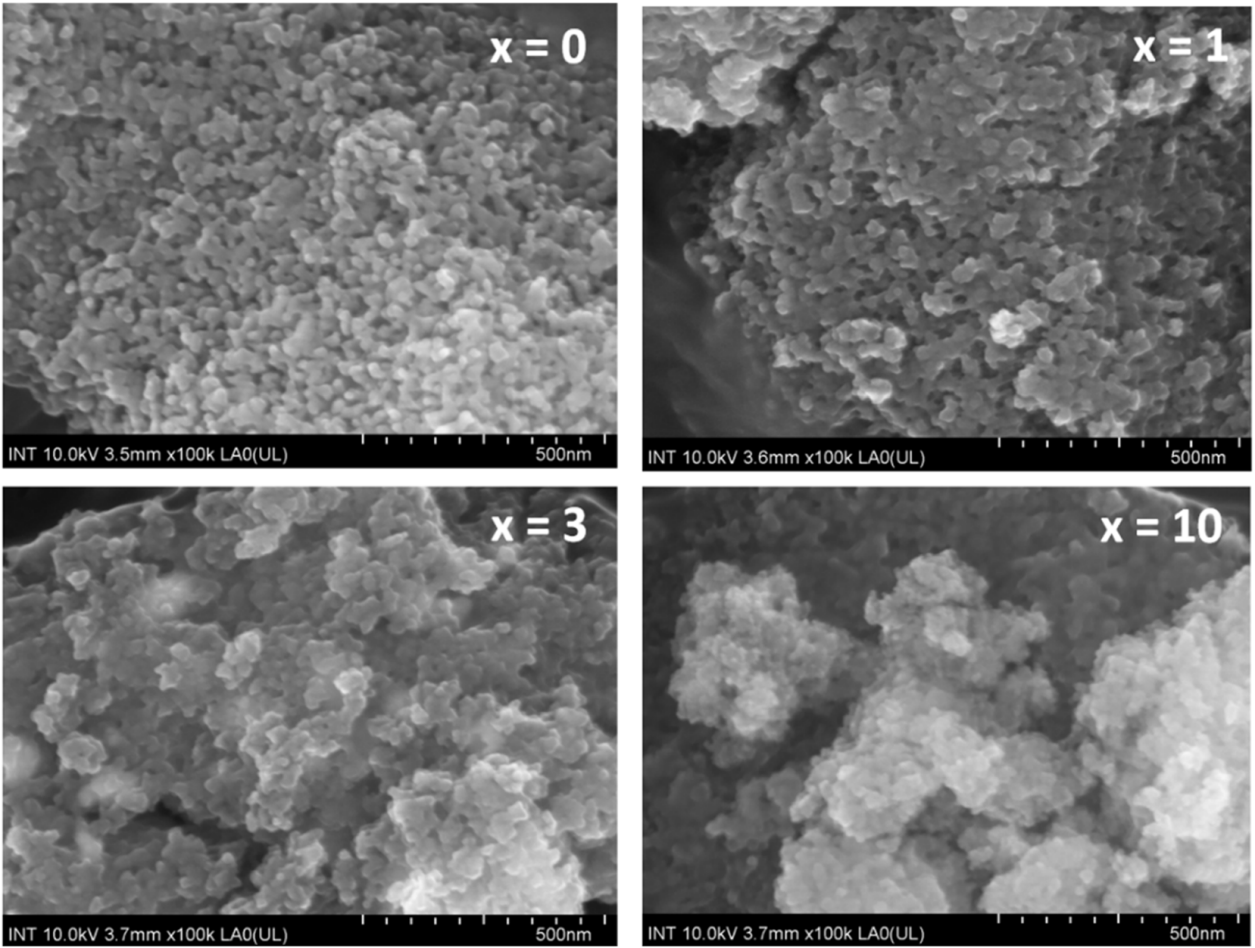
SEM observation of the 70SiO_2_–(26 − *x*)CaO–4P_2_O_5_–*x*Ag_2_O (*x* = 0, 1, 3, 10 mol%) samples.

Fig. 7 presents the SEM images of synthetic glasses 70SiO_2_–(26 − *x*)CaO–4P_2_O_5_–*x*Ag_2_O (*x* = 0, 1, 3, 10 mol%). The SEM image of the sample *x* = 0 showed the spherical nanoparticles, which are quite uniform. These nanoparticles are connected to each other to form an interconnected nano-porous structure. This result is much different from previous studies, where conventional sol–gel glasses often present a smooth surface or irregular structure.^27–29^ In this study, the modified sol–gel method based on the hydrothermal reaction with the vigorous conditions of temperature and self-pressure can cause the rapid generation of material particles with a fairly uniform structure. The SEM image of the sample *x* = 1 at the same magnification shows a quite obvious change compared to the sample *x* = 0. The uniform arrangement of nanoparticles is changed, it seems that the nanoparticles are agglomerated, making the surface of the material to become more sealed. The surface morphology is consistent with the above BET analysis, where the value of the specific surface area for sample *x* = 1 is reduced compared to sample *x* = 0. According to the previous research,^7^ probably the generated Ag nanoparticles fill up the pores making the surface structure denser. Thus, the specific surface area of the sample (*x* = 1) is lower than that of the sample *x* = 0. When the concentration of Ag_2_O gradually increases (sample *x* = 3, and *x* = 10), the surface structure of synthetic glass systems is strongly disturbed and separated into structural regions with larger porous gaps. This result is consistent with the above textural analysis, where samples *x* = 3, and *x* = 10 have increased the values of specific surface area and pore volume compared to sample *x* = 1.

**Fig. 8:**
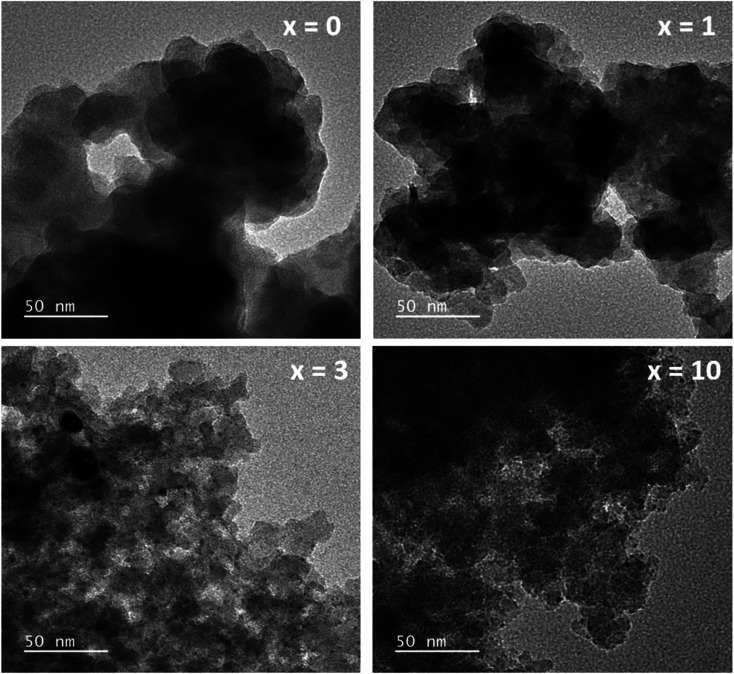
TEM observation of the 70SiO_2_–(26 − *x*)CaO–4P_2_O_5_–*x*Ag_2_O (*x* = 0, 1, 3, 10 mol%) samples.

The TEM images of glass samples are presented in Fig. 8. The sample without containing Ag (sample *x* = 0) shows the formation of nanoparticles with almost spherical shapes and sizes in the range between 20 and 30 nm. When adding Ag_2_O contents in the glass systems (sample *x* = 1, *x* = 3, *x* = 10), TEM images represent the deformation of initial nanoparticles and the decrease of the particle size. The decrease in the particle size could increase the specific surface area (sample *x* = 3, *x* = 10), which is also consistent with the previous study.^4^

**Fig. 9:**
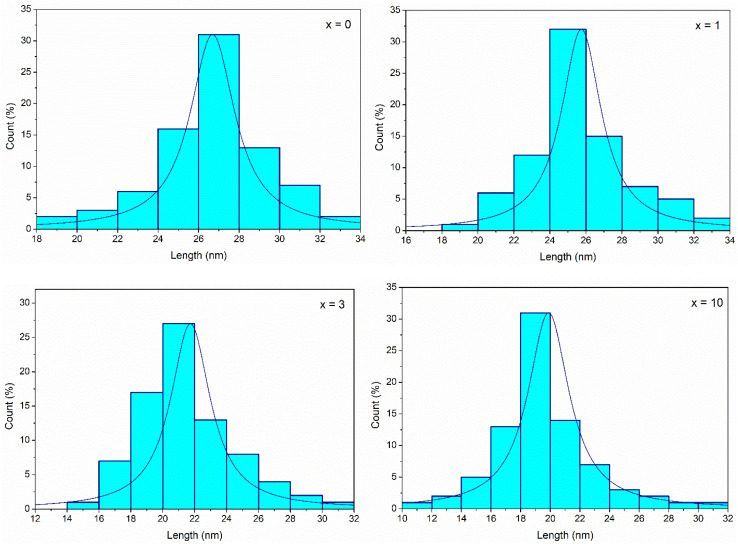
Particle size distributions of synthetic glasses 70SiO_2_–(26 − *x*)CaO–4P_2_O_5_–*x*Ag_2_O (*x* = 0, 1, 3, 10 mol%).

The histograms of particle size distributions of glasses 70SiO_2_–(26 − *x*)CaO–4P_2_O_5_–*x*Ag_2_O (*x* = 0, 1, 3, 10 mol%) obtained through Image-J analysis, are presented in Fig. 9. The average diameters of glass nanoparticles are 26.7; 25.7; 21.7; 19.8 nm, respectively for samples *x* = 0; *x* = 1; *x* = 3; and *x* = 10. Obviously, when increasing silver content, the glass particle size decreased which well matched with TEM image analysis.

### Bioactivity evaluation

**Fig. 10:**
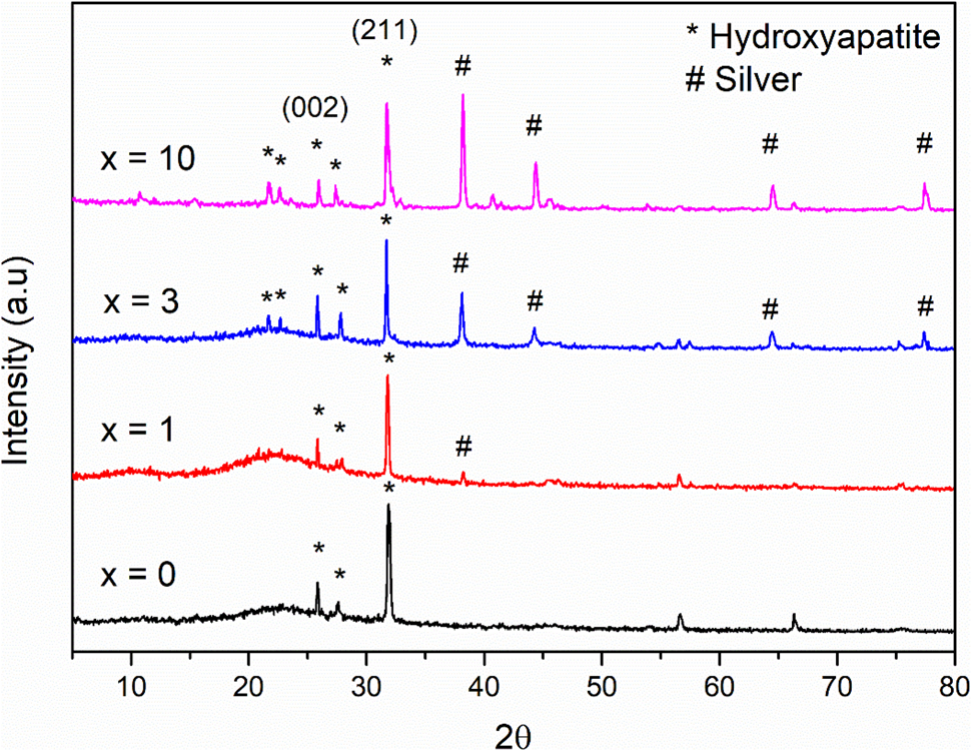
XRD patterns of the 70SiO_2_–(26 − *x*)CaO–4P_2_O_5_–*x*Ag_2_O (*x* = 0, 1, 3, 10 mol%) samples after 7 days of *in vitro* experiment.

The result of the XRD analysis of glass samples 70SiO_2_–(26 − *x*)CaO–4P_2_O_5_–*x*Ag_2_O (*x* = 0, 1, 3, 10 mol%) after *in vitro* experiments in SBF for 7 days is illustrated in Fig. 10. According to the standard JCPDS fiche [JCPDS-090432], the XRD patterns of all samples revealed two main peaks of hydroxyapatite (HA) mineral at 26°, and 32°corresponding to the (002), and (211) miller planes, respectively, which are confirmed the bioactivity of the synthetic glass systems. Additionally, crystalline peaks corresponding to HA are more clear when increasing the Ag_2_O contents in the glass systems (sample *x* = 0, *x* = 1 *versus* samples *x* = 3, *x* = 10). The sample (*x* = 3) exhibits the most obvious crystalline peak of HA, which well matches the above textural analysis. Therefore, when adding the content of Ag_2_O to the glass systems, the bioactivity of synthetic glass systems tends to increase because of the high specific surface area. This result is in high contrast to the previous studies.^7,10^ In this study, Ag-incorporated glass systems were synthesized by the modified sol–gel method based on the hydrothermal reaction in a non-acid-catalyzed environment. The factors such as shortened reaction time, the change of reaction medium, and vigorous reaction conditions (high temperature and self-pressure) strongly affect to structural morphology of the synthetic glass systems. The big difference in the synthetic method compared to the conventional sol–gel method makes the pore volume and specific surface area of most prepared bioactive systems only slightly change or even increase when increasing the Ag_2_O content. As a result, the bioactivity of synthetic glass systems tends to increase. On the other hand, in the previous studies, when increasing the content of Ag_2_O, the pore volume and specific surface area decreased significantly. Consequently, the bioactivity of synthetic glass systems tends to decrease rapidly.^6,9^ More specifically, silver crystalline peaks at 38°, 44°, 64°, and 77° appear more obvious and exhibit stronger intensity in synthetic samples with higher content of Ag_2_O (Fig. 2). After emerging in SBF solution, these diffraction patterns show much lower intensity than those before emerging. This decreased intensity of silver peaks is typical for the release of silver nanoparticles into the SBF medium due to the dissolution of the glass systems during *in vitro* experiment. Ag atoms released into the environment act as the antibacterial agent of the material.

**Fig. 11:**
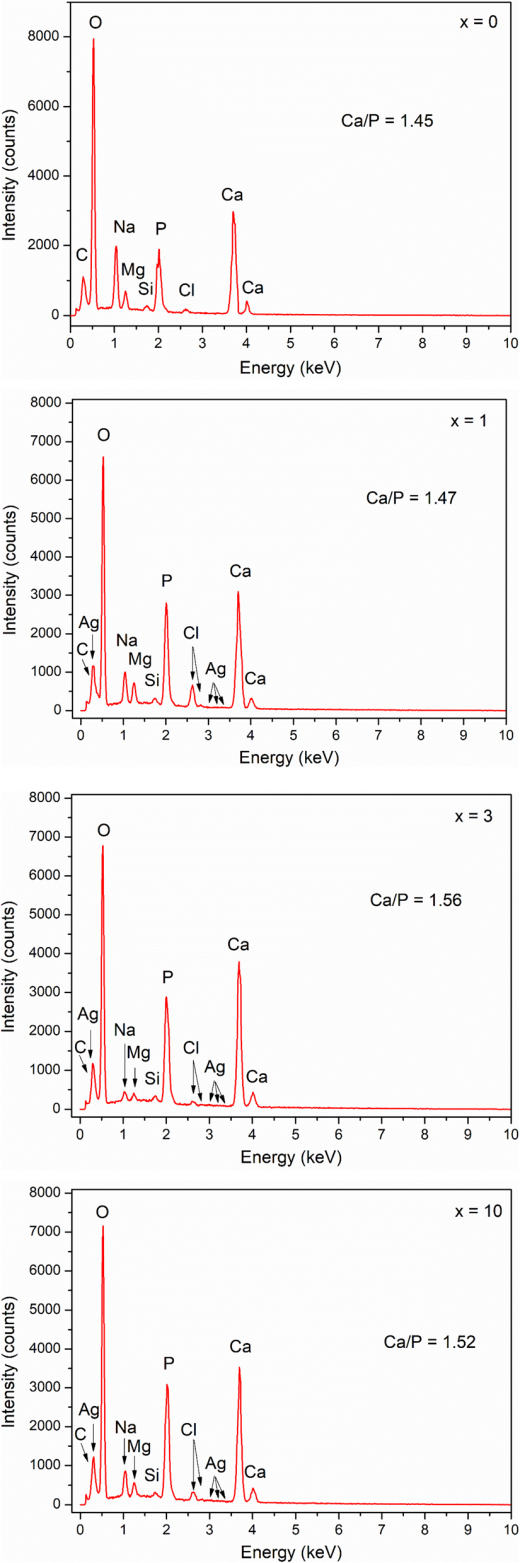
EDX analysis of glasses 70SiO_2_–(26 − *x*)CaO–4P_2_O_5_–*x*Ag_2_O (*x* = 0, 1, 3, 10 mol%) samples after 7 days of *in vitro* experiment.

The EDX method was used for the reference quantification of HA formed after *in vitro* experiments. Fig. 11 shows the elemental composition of the glass samples after 7 days of immersion in SBF solution. The Ca/P ratio was calculated for all samples. The Ca/P ratio increased from sample *x* = 0 to sample *x* = 3, then decreased slightly for sample *x* = 10. Sample *x* = 3 has a Ca/P ratio of 1.56, which is close to 1.67 of HA. Thus, the results of EDX and XRD analyses confirmed the bioactive behavior of synthetic glasses.

### Biocompatibility evaluation

**Fig. 12:**
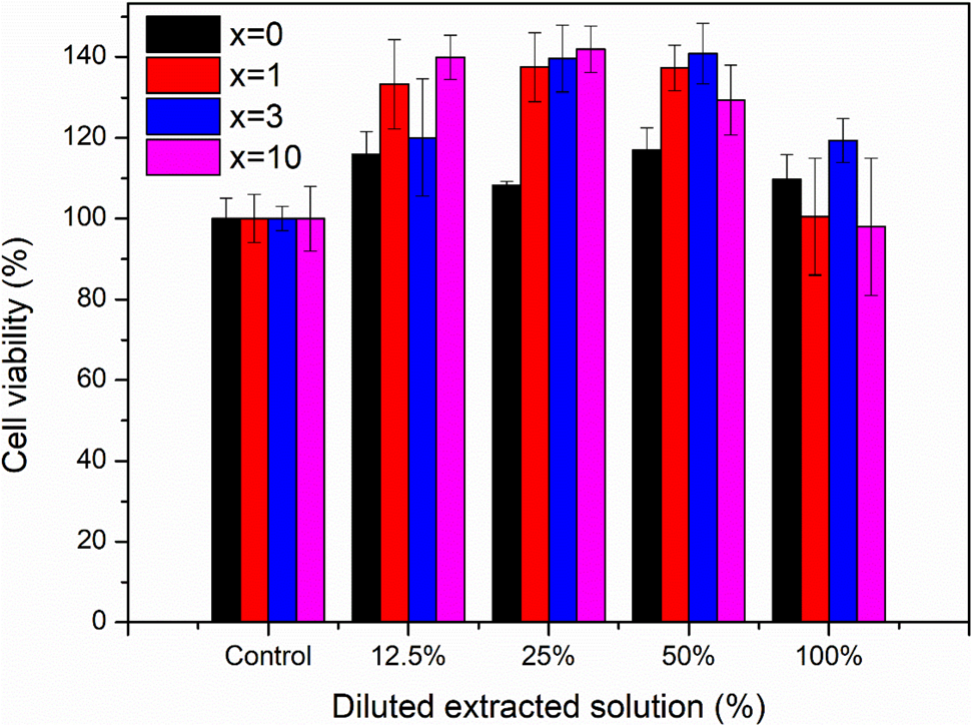
Cytotoxicity of synthetic bioactive glasses 70SiO_2_–(26 − *x*)CaO–4P_2_O_5_–*x*Ag_2_O (*x* = 0, 1, 3, 10 mol%).

**Fig. 13:**
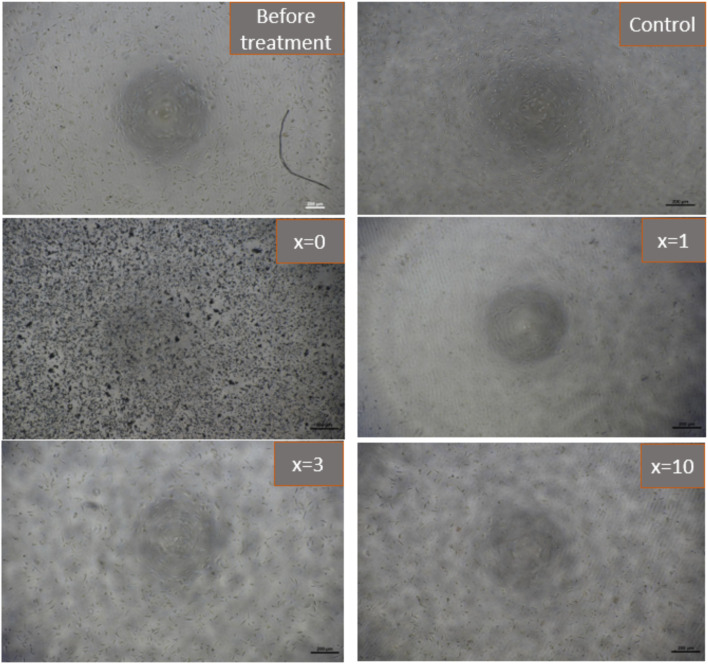
Images of fibroblasts cells (L-929) after exposure to synthetic bioactive glasses 70SiO_2_–(26 − *x*)CaO–4P_2_O_5_–*x*Ag_2_O (*x* = 0, 1, 3, 10 mol%) (100% extracted solution) for 24 hours.

Fig. 12 presents the results of the biocompatibility assessment of synthetic glasses by the MTT method. According to ISO 10993-5, these results indicate that the synthetic glasses are completely non-toxic, and have excellent biocompatibility, allowing for cell viability. Cell survival was higher than 90% for all concentrations of extracts. The extracts with low concentrations (12.5, 25, 50%) all showed cell survival ability was higher than 100%. With Ag-incorporated glasses containing 10% mol of Ag_2_O, cell viability tends to decrease, but it is still higher than the safe value for cell viability (70% according to ISO 10993-5). The images of fibroblast cells after exposure to the glass extract at the highest concentration (100%) are selected as a representative (Fig. 13). The images showed the good survival of cells when exposed to the glass extract. The obtained cytotoxicity evaluation results are quite similar to the previous study,^20,21^ confirming the biocompatibility of Ag-incorporated glasses.

### Antibacterial test

**Fig. 14:**
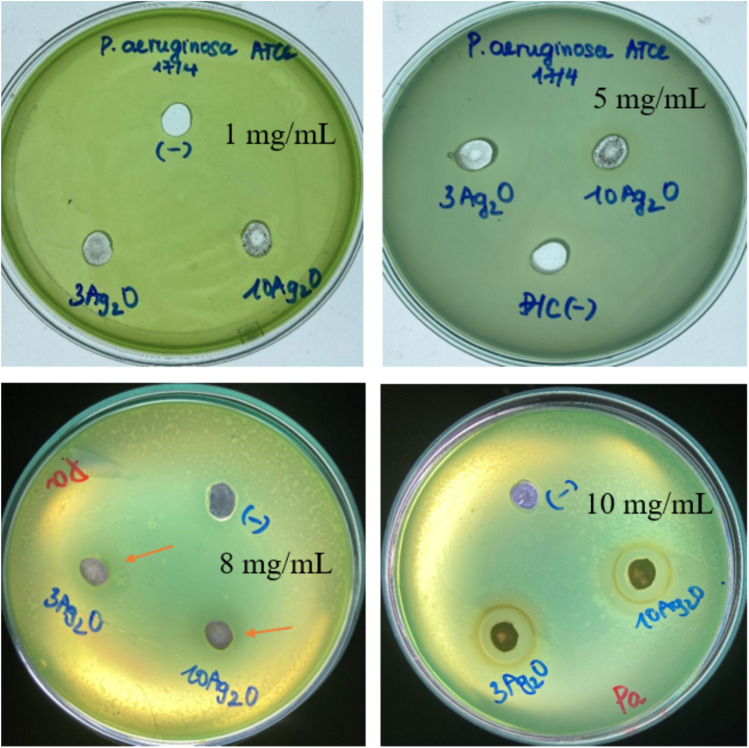
Diameters of antibacterial zones of glass samples 70SiO_2_–(26 − *x*)CaO–4P_2_O_5_–*x*Ag_2_O (*x* = 3, 10 mol%) (sample *x* = 3, and *x* = 10) for *Pseudomonas aeruginosa* (Pa).

According to the previous study, released silver can kill bacteria by attaching to DNA, and RNA, and binding to proteins and their membranes, thereby inhibiting bacterial respiration.^33,34^ The antibacterial activities of selected glass samples (sample *x* = 3, noted as 3Ag_2_O; and sample *x* = 10, noted as 10Ag_2_O) are presented in Fig. 14.

The results showed that the experimental plates did not produce antibacterial inhibition zones for samples with low glass concentrations (samples containing 1 and 5 mg mL^−1^ of glass powder). The test plate with a concentration of 8 mg mL^−1^ showed a fairly broad inhibition zone, but it is not clear. The test plate with a concentration of 10 mg mL^−1^ showed an obvious zone of bacterial inhibition. Therefore, Ag-doped glass clearly shows antibacterial ability at the test concentration of 10 mg mL^−1^. The diameters of inhibition zones for the sample *x* = 3, and the sample *x* = 10 are about 16 ± 0.5 mm, and 15 ± 0.5 mm, respectively. The obtained data show that the sample containing less Ag_2_O (sample *x* = 3) has a slightly higher antibacterial ability than the sample containing more Ag_2_O (sample *x* = 10). This could be explained by the different structural morphology of the synthesized samples affecting the ability to release metallic silver nanoparticles in a bacterial culture medium. The release of silver metal of sample *x* = 3 is better than that of sample *x* = 10, probably due to slightly higher pore volume and specific surface area (Table 6). Consequently, the bacterial inhibition of sample *x* = 3 is slightly stronger than that of sample *x* = 10.

## Conclusion

For the first time, Ag-incorporated bioactive glasses were successfully synthesized by the modified sol–gel method based on the hydrothermal reaction. The synthesis time was shortened, and the synthesis process did not use acid or base catalysts compared to the conventional sol–gel method. The Ag-incorporated glasses are mesoporous structures, which exhibit much higher pore volume and specific surface area values than those of similar glasses synthesized by the conventional sol–gel method. The bioactivity of synthetic glasses was confirmed by the formation of apatite mineral after *in vitro* experiment. Especially, the synthetic glasses in this study did not show a decrease in bioactivity when silver was added. The Ag-incorporated glasses show good biocompatibility with fibroblast cells (L-929). The antibacterial activity of Ag-incorporated glass samples was confirmed against the bacterial strain *Pseudomonas aeruginosa* by producing distinct antibacterial rings. Thus, the glass systems in this study exhibit bioactive, biocompatible, and antibacterial properties. Furthermore, the glass systems were synthesized by non-catalyzed green synthesis, which could lead to new applications such as the addition of glasses in smart toothpaste, or cosmetics.

## Conflicts of interest

The authors declare that they have no conflict of interest in this work.

## Supplementary Material
